# *In vitro* pharmacokinetics/pharmacodynamics of the β-lactamase inhibitor, durlobactam, in combination with sulbactam against *Acinetobacter baumannii-calcoaceticus* complex

**DOI:** 10.1128/aac.00312-23

**Published:** 2023-12-11

**Authors:** John O'Donnell, Angela Tanudra, April Chen, Alita A. Miller, Sarah M. McLeod, Rubén Tommasi

**Affiliations:** 1 Entasis Therapeutics, Inc., Waltham, Massachusetts, USA; Providence Portland Medical Center, Portland, Oregon, USA

**Keywords:** durlobactam, sulbactam, pharmacokinetics, pharmacodynamics, *Acinetobacter calcoaceticus*

## Abstract

Infections caused by *Acinetobacter baumannii* are increasingly multidrug resistant and associated with high rates of morbidity and mortality. Sulbactam is a β-lactamase inhibitor with intrinsic antibacterial activity against *A. baumannii*. Durlobactam is a non-β-lactam β-lactamase inhibitor with an extended spectrum of activity compared to other inhibitors of its class. *In vitro* pharmacodynamic infection models were undertaken to establish the pharmacokinetic/pharmacodynamic (PK/PD) index and magnitudes associated with sulbactam and durlobactam efficacy and to simulate epithelial lining fluid (ELF) exposures at clinical doses to understand sulbactam-durlobactam activity with and without co-administration of a carbapenem. Hollow fiber infection models (HFIMs) and one-compartment systems were used to identify the PK/PD indices and exposure magnitudes associated of 1-log_10_ and 2-log_10_ colony-forming unit (CFU)/mL reductions. Sulbactam and durlobactam demonstrated PK/PD drivers of % time above the minimum inhibition concentration (%T > MIC) and area under the plasma concentration-time curve from time 0 to 24 h (AUC_0–24_)/MIC, respectively. Against a sulbactam-susceptible strain, sulbactam %T > MIC of 71.5 and 82.0 were associated with 1-log_10_ and 2-log_10_ CFU/mL reductions, respectively, in the HFIM. Against a non-susceptible strain, durlobactam restored the activity of sulbactam with an AUC_0–24_/MICs of 34.0 and 46.8 using a polysulfone cartridge to achieve a 1-log_10_ and 2-log_10_ CFU/mL reduction. These magnitudes were reduced to 13.8 and 24.2, respectively, using a polyvinylidene fluoride cartridge with a membrane pore size of 0.1 μm. In the one-compartment model, durlobactam AUC_0–24_/MIC to achieve 1-log_10_ and 2-log_10_ CFU/mL reduction were 7.6 and 33.4, respectively. Simulations of clinical ELF exposures in the HFIM showed cidal activity at MICs ≤4 µg/mL. Penicillin binding protein 3 mutant strains with MICs of 8 μg/mL may benefit from the addition of a carbapenem at clinical exposures.

## INTRODUCTION

Multidrug-resistant (MDR) *Acinetobacter baumannii* are pathogens involved in serious nosocomial infections with increasing prevalence and incidence of antibiotic resistance, including resistance to carbapenems (1 – 3). These infections are often associated with high rates of morbidity and mortality (1, 4), and effective treatments are limited (5). Carbapenem-resistant *A. baumannii* (CRAB) has been identified as a global threat with an urgent unmet medical need (6, 7). With the need to discover and develop new therapies, β-lactam-β-lactamase inhibitor combinations provide important options to address the threat posed by β-lactamase-producing bacteria including *A. baumannii*.

Sulbactam is a first-generation, narrow-spectrum β-lactamase inhibitor used in combination with ampicillin as the marketed product Unasyn. Interestingly, sulbactam has intrinsic antibacterial activity against *Acinetobacter* spp. due to its ability to inhibit penicillin binding protein 1 and penicillin binding protein 3 (PBP3) which are essential for cell wall synthesis of Gram-negative bacteria (8). This attribute has led to the use of ampicillin-sulbactam in the treatment of *A. baumannii* infections. The activity of sulbactam, however, has diminished against contemporary isolates of *A. baumannii* which demonstrate decreased susceptibility due to β-lactamase-mediated resistance (9, 10).

Durlobactam is a non-β-lactam β-lactamase inhibitor with an extended spectrum of activity compared to other β-lactamase inhibitors (9). *In vitro*, durlobactam potently inhibits Ambler class A, C, and D β-lactamases, although it has no significant intrinsic antibacterial activity against *A. baumannii* on its own (10). Importantly, durlobactam inhibits class D carbapenemases of the OXA family, which are some of the prevailing mechanisms of carbapenem resistance in *A. baumannii*. Results from *in vitro* and *in vivo* studies indicate that durlobactam protects sulbactam from hydrolysis by β-lactamases and restores its activity against carbapenem-resistant and MDR *A. baumannii* (9). In a study of 4,038 recent, global, clinical isolates of *A. baumannii*, when durlobactam was added to sulbactam *in vitro*, the MIC_90_ of sulbactam decreased from 64 µg/mL to 2 µg/mL (11). Additional studies have corroborated these findings of potent *in vitro* activity against carbapenem-resistant *A. baumannii* isolates (12 – 14). Previous pharmacokinetic/pharmacodynamic (PK/PD) and efficacy studies completed using *in vitro* and *in vivo* models of infection with susceptible *A. baumannii* strains suggest that sulbactam activity correlates to unbound time above MIC (*f*T > MIC) (15, 16). The present study seeks to determine the PK/PD indices and exposures associated with the activity of sulbactam and durlobactam when used in combination against multidrug-resistant (MDR) *A. baumannii* using *in vitro* pharmacodynamic infection models.

## RESULTS

### *In vitro* susceptibility testing

Studies were performed to establish the optimal *in vitro* susceptibility test method for sulbactam-durlobactam. Several sulbactam:durlobactam ratios as well as titrations of sulbactam in the presence of different fixed concentrations of durlobactam were assayed for their ability to discriminate between *A. baumannii* isolates that should be susceptible or resistant to the sulbactam-durlobactam combination based on resistance elements encoded by each isolate. Titration of sulbactam in the presence of a fixed concentration of 4 µg/mL durlobactam most accurately separated the predicted susceptible and predicted resistant isolates (17). MIC values of sulbactam and sulbactam in the presence of 4 µg/mL of durlobactam are summarized in Table 1 for the strains used in this study. One of the isolates in this study was susceptible to sulbactam alone (MIC = 2 µg/mL) based on the Clinical and Laboratory Standards Institute (CLSI) breakpoint of 8/4 µg/mL for the 2:1 ampicillin:sulbactam combination, where sulbactam is the component that confers activity against *Acinetobacter* spp. (CLSI M100). The other four isolates (ARC3486, ARC5081, ARC5950, and ARC5955) had sulbactam MIC values of ≥16 µg/mL and were also carbapenem resistant as shown in Table 1. When 4 µg/mL durlobactam was added to sulbactam, the MIC values for the sulbactam-resistant isolates dropped ≥4-fold with MICs ranging from 0.5 to 8 µg/mL. Two isolates had sulbactam-durlobactam MICs of 8 µg/mL (ARC5950 and ARC5955), which corresponds to an intermediate susceptibility interpretation based on FDA breakpoints (18), and both encoded for single amino acid substitutions near the active site of PBP3, the target of sulbactam antibacterial activity (8).

**TABLE 1:** MIC summary of *A. baumannii* isolates used for *in vitro* time kill and PK/PD studies

Isolate	Genotype	MIC (µg/mL)^*a*^			
		SUL	SUL + DUR^*b*^	SUL:IMP (1:1) + DUR^*c*^	SUL:MEM (1:1) + DUR^*d*^
ARC2058	ADC-99 [N379S]; OXA-259	2	1	ND	ND
ARC3486	ADC-30; TEM-1; OXA-66; OXA-72	32	0.5	0.5	0.5
ARC5081	ADC-80; ADC-176; OXA-23; OXA-94	16	4	1	2
ARC5950	ADC-11; OXA-23, OXA-69; PBP3 [T526S]	64	8	4	4
ARC5955	ADC-82; TEM-1; OXA-23; OXA-66; PBP3 [P508A, A515T]	64	8	8	8

^*a*^ Modal MIC.

^*b*^ MIC of sulbactam in the presence of 4 µg/mL durlobactam.

^*c*^ MIC of 1:1 sulbactam:imipenem in the presence of 4 µg/mL durlobactam.

^*d*^ MIC of 1:1 sulbactam:meropenem in the presence of 4 µg/mL durlobactam.

Because sulbactam-durlobactam was dosed on a background of imipenem in the phase 3 trial to treat co-infecting, non-*Acinetobacter* pathogens (19), the ARC3486, ARC5950, and ARC5955 were tested for susceptibility to triple combinations of either imipenem or meropenem and sulbactam-durlobactam. This was assayed as a titration of a 1:1 ratio of sulbactam and carbapenem in the presence of a fixed concentration of 4 µg/mL durlobactam. The MICs for the triple combination were the same as for sulbactam-durlobactam for ARC3486 and ARC5950, which are 0.5 and 8 µg/mL, respectively. For ARC5955, the MIC of imipenem or meropenem in the presence of sulbactam-durlobactam was 4 µg/mL, which was twofold dilution lower than was seen for sulbactam-durlobactam.

### Sulbactam and durlobactam concentrations

**Fig 1:**
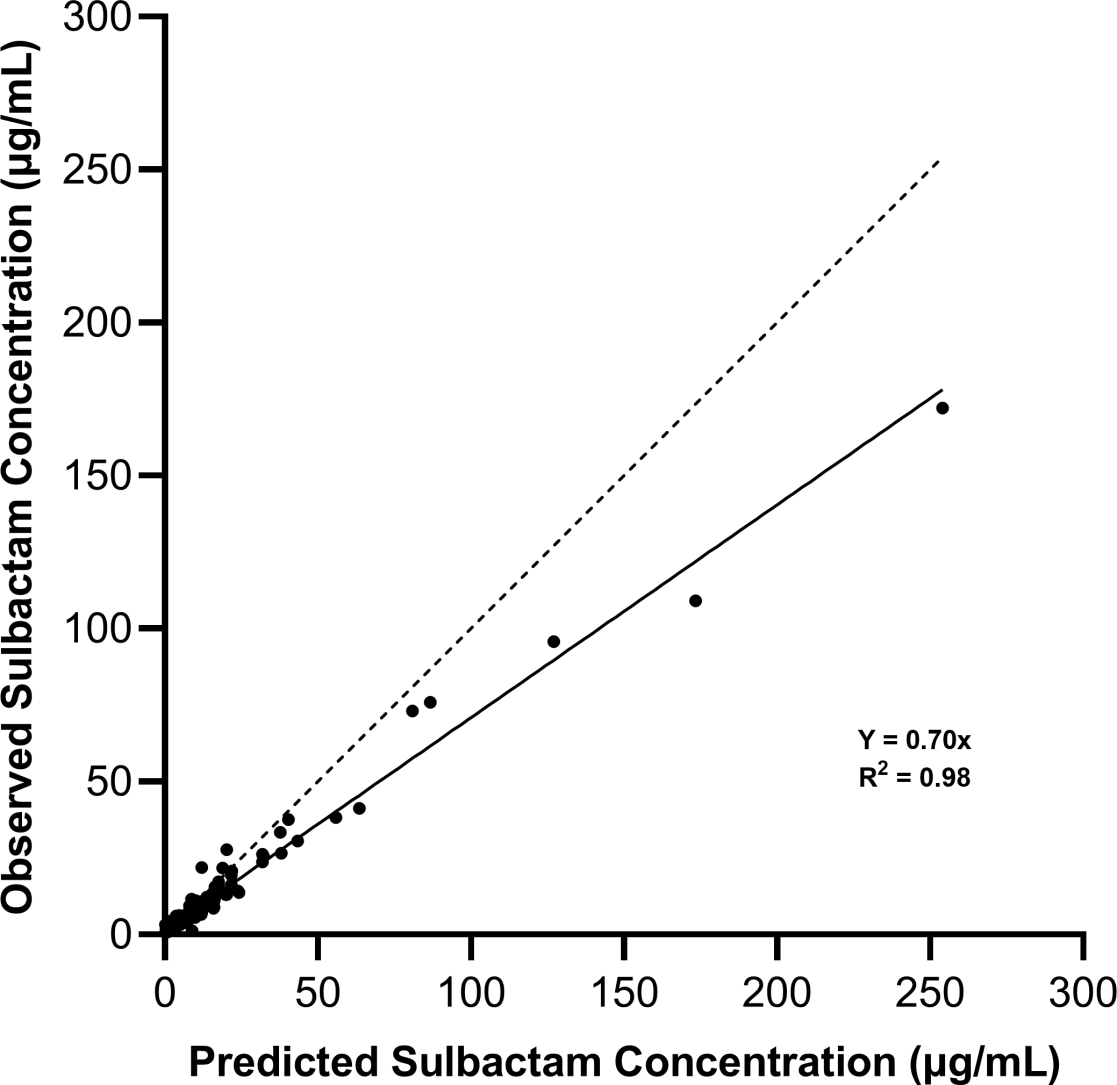
Observed vs predictive concentration plot of sulbactam in the hollow fiber infection model vs *A. baumannii* ARC2058 (polysulfone cartridge).

**Fig 2:**
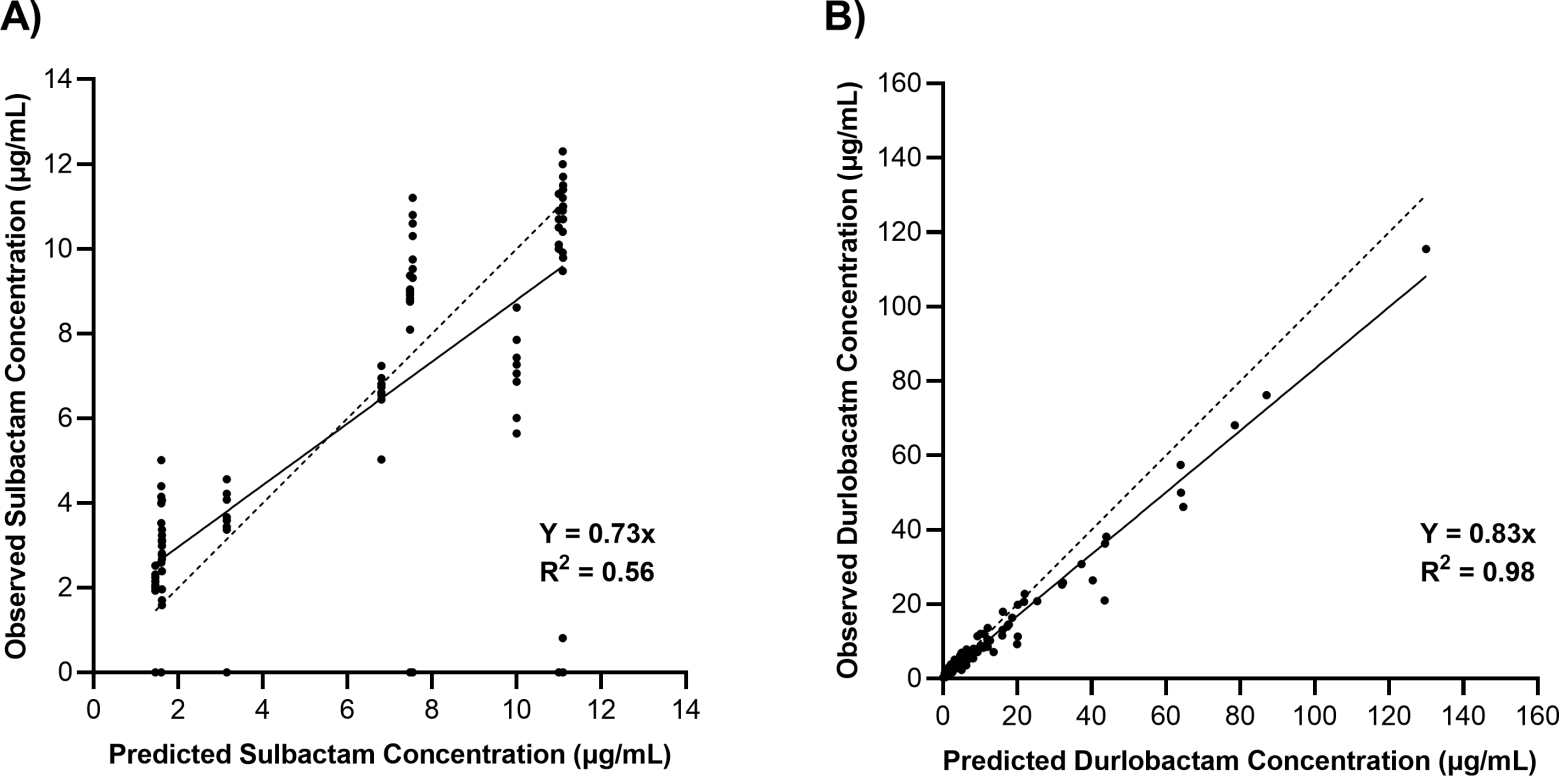
Observed vs predictive concentration plot of sulbactam (**A**) and durlobactam (**B**) in the hollow fiber infection model vs *A. baumannii* ARC5081 (polysulfone cartridge).

**Fig 3:**
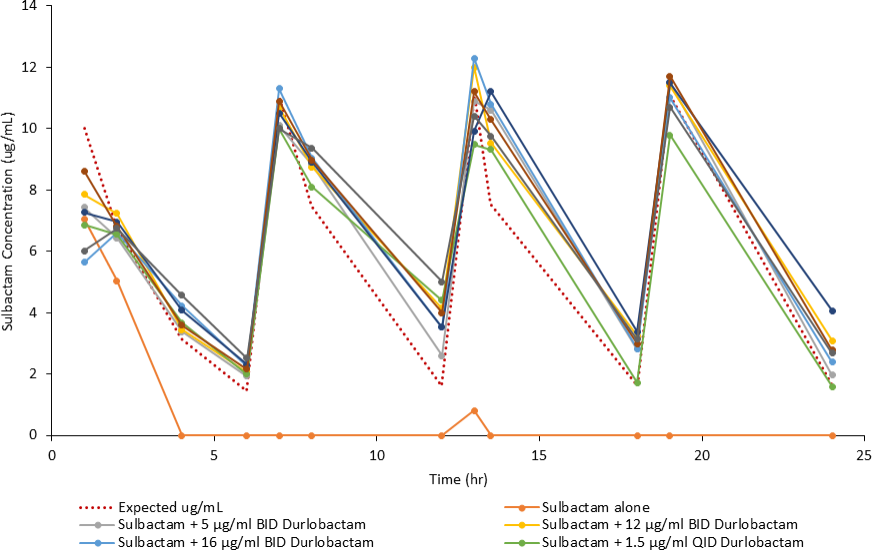
PK sampling of sulbactam (q6h) with and without durlobactam regimens vs *A. baumannii* ARC5081 (1-h infusion).

Concentrations of sulbactam and durlobactam were determined by liquid chromatography-tandem mass spectrometry (LC/MS/MS) and utilized directly for assessing exposure in samples obtained over the 24-h studies performed in the hollow fiber infection models (HFIMs) and one-compartment model systems. Observed vs predicted concentration data of sulbactam vs the sulbactam-sensitive isolate ARC2058 in an HFIM utilizing polysulfone cartridges are shown in Fig. 1. Polysulfone cartridges are commonly used in the HFIM, but with a molecular weight cut-off of 20 kDa, accumulation of enzymes such as β-lactamases may occur. Generally, lower concentrations of sulbactam were observed relative to predicted exposures (Fig. 1). Observed vs predicted concentrations of sulbactam vs ARC5081 with and without durlobactam are shown in Fig. 2A, and durlobactam observed vs predicted concentrations are shown in Fig. 2B. Evidence of sulbactam degradation by the second dose vs ARC5081 was readily observed in the sulbactam-only treatment arm (Fig. 3). In the HFIM studies vs ARC5081 using a polyvinylidene fluoride (PVDF) cartridges with a 0.1-µm pore size and one-compartment model systems, sulbactam and durlobactam observed vs predicted concentrations were generally in good agreement (Fig. S1 and S2). All PK/PD assessments involving exposure response analyses were completed using the observed (assayed) concentrations of sulbactam and durlobactam.

### Sulbactam and durlobactam *in vitro* dose fractionation studies in a hollow fiber infection model (polysulfone cartridge)

**Fig 4:**
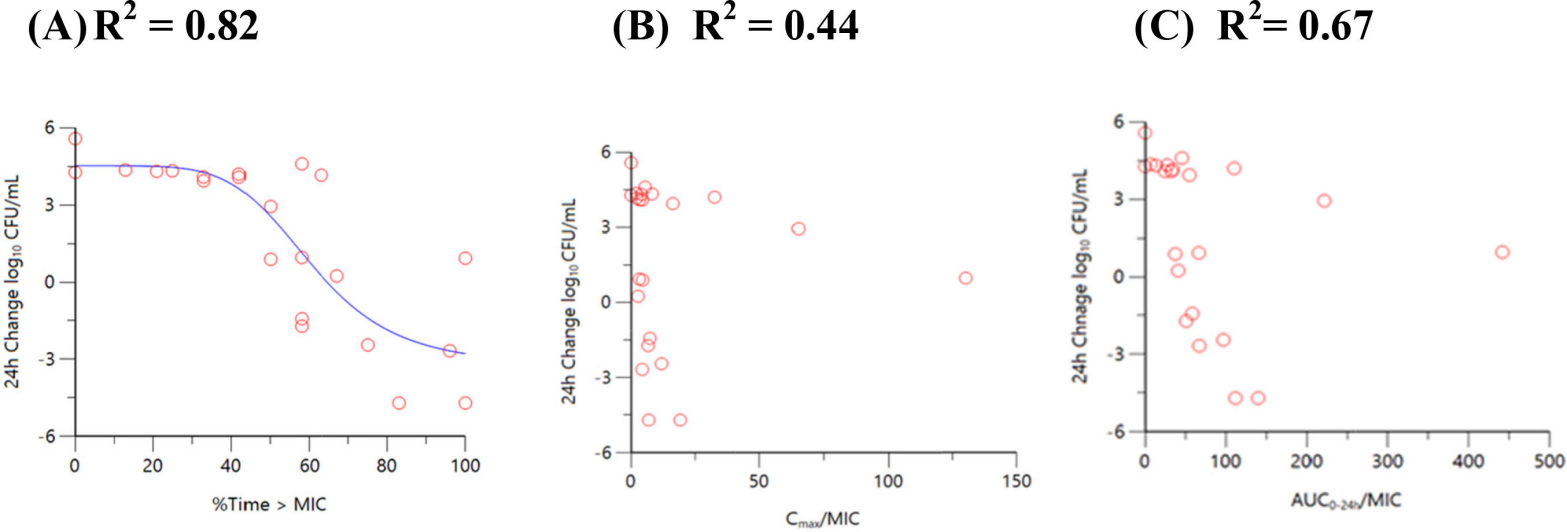
Relationship between sulbactam (**A**) time above MIC, (**B**) C_max_/MIC, and (**C**) AUC_0–24_/MIC vs *A. baumannii* ARC2058 response in the HFIM (polysulfone cartridges).

Dose fractionation studies of sulbactam (alone) vs a sulbactam-sensitive strain, *A. baumannii* ARC2058, were performed in the HFIM with polysulfone cartridges. Sulbactam was administered via a 1-h infusion. As shown in Fig. 4, time above MIC best described the relationship between change in log_10_ colony-forming unit (CFU)/mL from baseline at 24 h and sulbactam exposure with a correlation coefficient (*R* ^2^) value of 0.82. As summarized in Table 2, the sulbactam % time above the minimum inhibition concentration (%T > MIC) associated with 1- and 2-log_10_ CFU/mL reductions were 71.5 and 82.0, vs *A. baumannii* ARC2058, respectively.

**TABLE 2:** PK/PD endpoint exposures associated with activity of sulbactam and durlobactam in a hollow fiber infection model and a one-compartment model

Compound (index) model	Strain	PK/PD endpoint		
		CFU reduction from baseline		EC_80_
		1-log_10_	2-log_10_	
Sulbactam (T > MIC)				
HFIM (polysulfone)	ARC2058	71.5%	82.0%	93.6%
Durlobactam (AUC_0–24_/MIC)				
HFIM (polysulfone)	ARC5081	34.0	46.9	NC
HFIM (PVDF 0.1-µm pore)	ARC5081	13.8	24.2	NC
One-compartment model	ARC5081	7.6	33.4	NC

**Fig 5:**
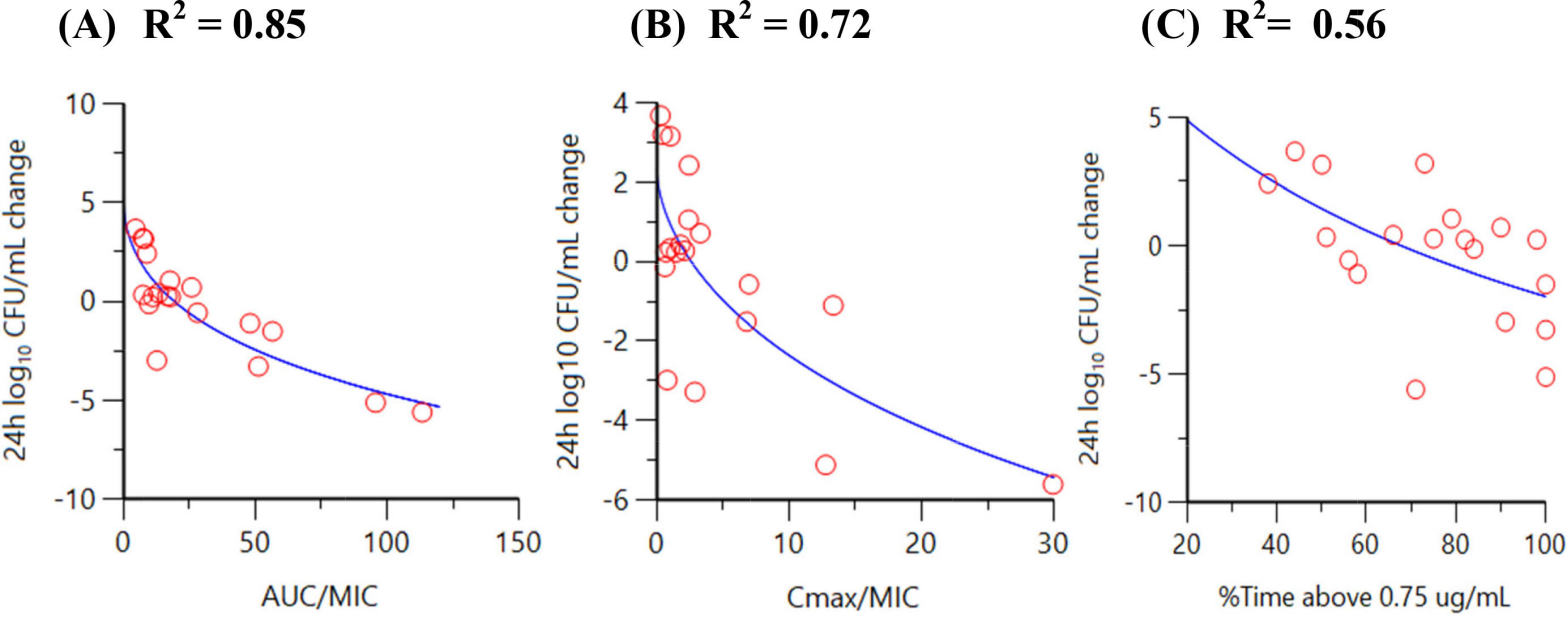
Relationship between durlobactam AUC/MIC (**A**), C_max_/MIC (**B**), and %Time > critical threshold value of 0.75 µg/mL (**C**) vs *A. baumannii* ARC5081 response in the HFIM (polysulfone cartridges).

In the presence of a regimen of sulbactam administered every 6 h (q6h) with a targeted maximum (peak) plasma concentration (C_max_) of 12 µg/mL, dose fractionation of durlobactam via regimens of q6h, every 12 h (q12h), and every 24 h (q24h) was completed to determine the PK/PD driver of durlobactam vs the CRAB isolate ARC5081. This was initially completed using a 1-h infusion of both compounds; however, given the evidence of sulbactam degradation in the initial durlobactam dose fractionation (Fig. 3), the infusion was switched to 3 h for both compounds resulting in T > MIC of 85% for the sulbactam q6h regimen. As shown in Fig. 5, the PK/PD index that demonstrated the greatest correlation to the activity of durlobactam vs *A. baumannii* ARC5081 was the area under the plasma concentration-time curve from time 0 to 24 h (AUC_0–24_)/MIC with an *R* ^2^ value of 0.85. AUC_0–24_/MIC ratios of 34.0 and 46.8 were associated with achieving 1-log_10_ and 2-log_10_ CFU reduction, respectively (Table 2). Correlation coefficients vs C_max_/MIC, AUC_0–24_/MIC, and T > C_T_ of 0.5 to 4 µg/mL are summarized in Table S1. Bacterial colonies did not demonstrate reduced susceptibility at 24 h when plated on sulbactam-durlobactam plates at 3× MIC.

### Durlobactam *in vitro* dose fractionation studies in a hollow fiber infection model (PVDF cartridge)

Based upon the observation of less than predicted concentrations determined for sulbactam in the polysulfone cartridges, dose fractionation experiments were performed in PVDF cartridges with a 0.1-µm pore size to allow for larger molecules such as β-lactamases to pass through the hollow fiber membranes and exchange with the extra-capillary space.

**Fig 6:**
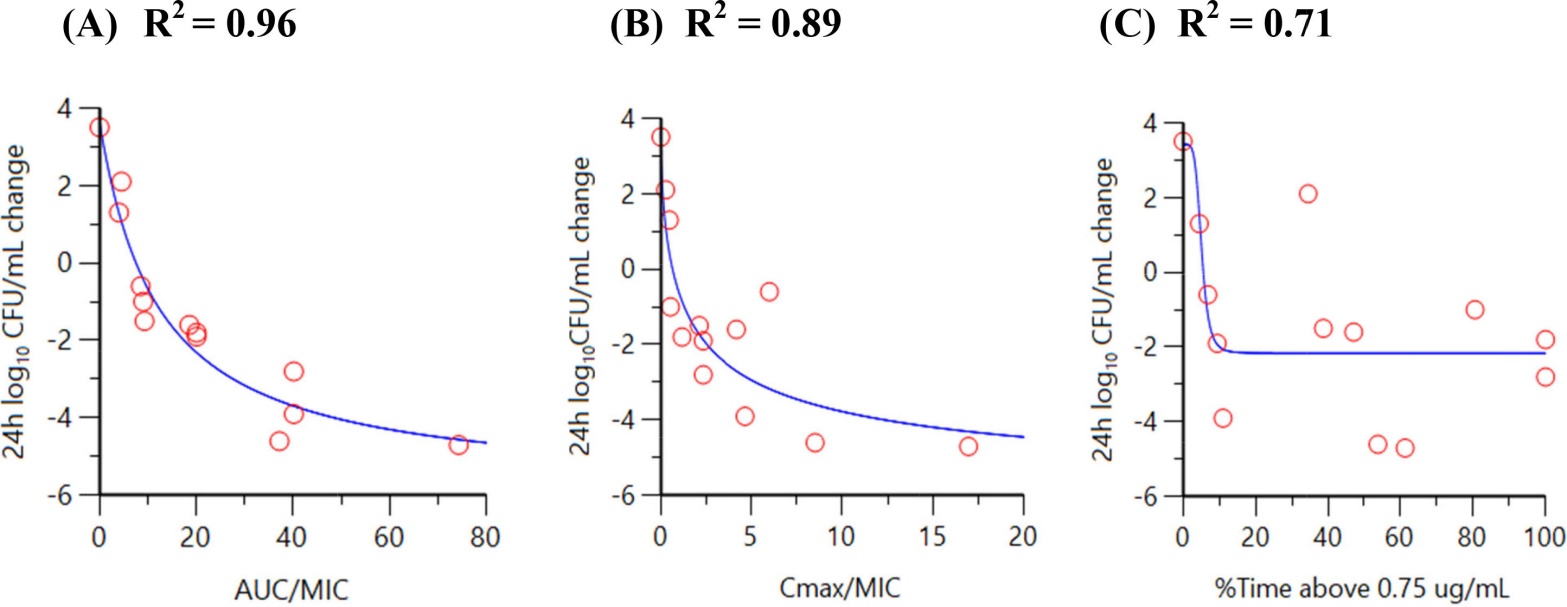
Relationship between durlobactam AUC/MIC (**A**), C_max_/MIC (**B**), and %Time > critical threshold value of 0.75 µg/mL (**C**) vs *A. baumannii* ARC5081 response in the HFIM (PVDF cartridges).

In the presence of a q6h regimen of sulbactam with a targeted C_max_ of 12 µg/mL following a 3-h infusion (%T > MIC ~85% observed), dose fractionation of durlobactam via q6h, q12h, and q24h regimens was completed to determine the PK/PD driver of durlobactam vs the CRAB isolate ARC5081 using the PVDF cartridges. As shown in Fig. 6, the PK/PD index that demonstrated the greatest correlation to the activity of durlobactam vs *A. baumannii* ARC5081 was AUC_0–24_/MIC with an *R* ^2^ value of 0.95. AUC_0–24_/MIC ratios of 13.8 and 24.2 were associated with achieving 1-log_10_ and 2-log_10_ CFU reduction, respectively (Table 2). Correlation coefficients vs C_max_/MIC, AUC_0–24_/MIC, and T > C_T_ of 0.5 to 4 µg/mL are summarized in Table S2. Bacterial colonies did not demonstrate reduced susceptibility at 24 h when plated on sulbactam-durlobactam plates at 3× MIC.

### Durlobactam *in vitro* dose fractionation study in a one-compartment model

**Fig 7:**
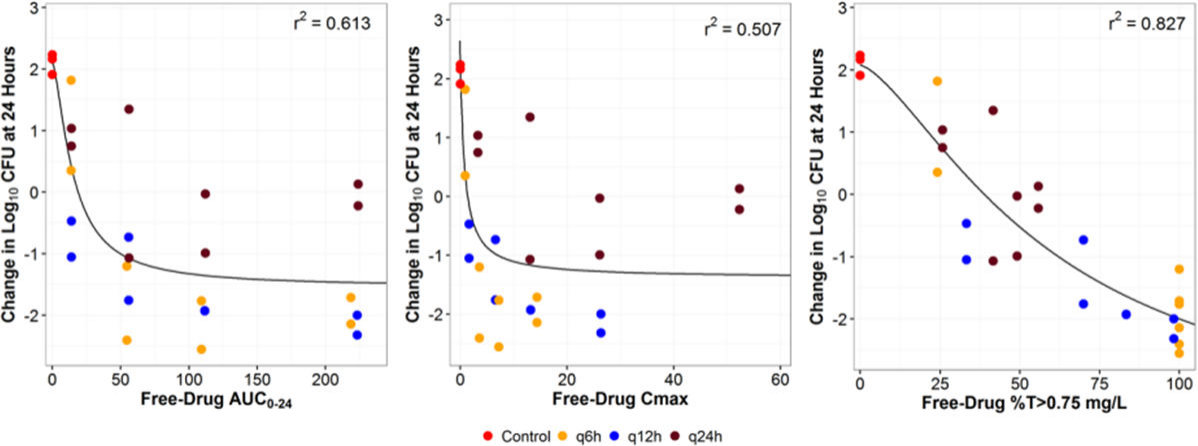
Relationship between durlobactam AUC_0–24_, C_max_, and %T > durlobactam threshold value of 0.75 µg/mL against *A. baumannii* ARC5081 response in the one-compartment model (color coded by regimen). %T > 0.75 µg/mL, time as a percentage of the dosing interval that the drug concentration remains above 0.75 µg/mL; AUC_0–24_, area under the plasma concentration-time curve from time 0 to 24 h; CFU, colony-forming units; C_max_, maximum (peak) plasma concentration; *r* ^2^, correlation coefficient.

**Fig 8:**
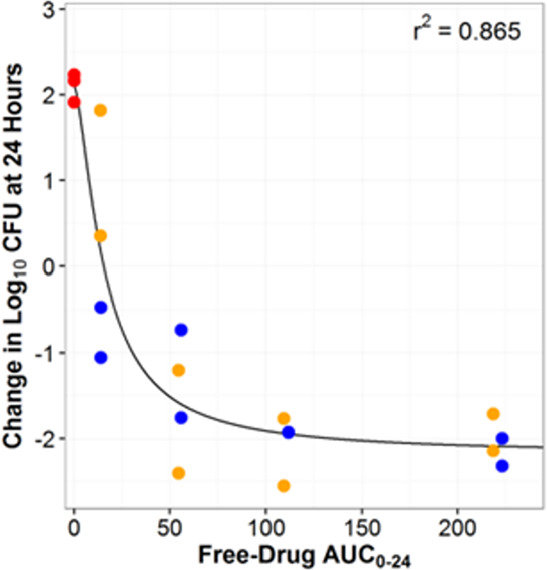
Relationship between the change in log_10_ CFU/mL from baseline and durlobactam AUC_0-24_ for *A. baumannii* ARC5081 examined in dose fractionation studies (q6h and q12h data only) AUC_0–24_, area under the plasma concentration-time curve from time 0 to 24 h; CFU, colony-forming units; C_max_, maximum (peak) plasma concentration; *r* ^2^, correlation coefficient.

Results of dose fractionation studies of durlobactam vs a sulbactam-resistant strain, *A. baumannii* ARC5081, performed in a one-compartment *in vitro* infection model are shown in Fig. 7. Against *A. baumannii* ARC5081, when used in combination with sulbactam, time above a critical threshold (T > C_T_) of 0.75 µg/mL best described the relationship between the change in log_10_ CFU/mL from baseline at 24 h and durlobactam exposure with an *R* ^2^ of 0.827. Durlobactam C_max_ and AUC_0–24_ exposures demonstrated lower *R* ^2^ of 0.507 and 0.613, respectively. Utilizing only q6h and q12h data from the one-compartment model, the relationship between the change in log_10_ CFU/mL from baseline at 24 h and AUC_0–24_ is summarized in Fig. 8. The *R* ^2^ for AUC_0–24_ vs the 24-h change in log_10_ CFU/mL improved from 0.613 to 0.865 and was subsequently selected to normalize by MIC and use as a PK/PD target based upon the collective PK/PD driver result for the durlobactam in the HFIM and analysis of the one-compartment data. AUC_0–24_ values of 30.5 µg·h/mL and 134 µg·h/mL were associated with a 1-log_10_ and 2-log_10_ CFU reduction from baseline over 24 h, respectively. Using the modal MIC of 4 µg/mL for ARC5081, these targets further correspond to AUC_0–24_/MIC ratios of 7.6 and 33.4 for a 1-log_10_ and 2-log_10_ CFU reduction from baseline, respectively (Table 2), which were lower than AUC_0–24_/MIC ratios observed in the HFIM studies using polysulfone cartridges and more consistent with the HFIM studies utilizing the PVDF cartridges. Bacterial colonies did not demonstrate reduced susceptibility at 24 h when plated on sulbactam-durlobactam plates at 3× MIC.

### Clinical regimen testing of sulbactam in combination with durlobactam in hollow fiber infection model with and without a carbapenem

**Fig 9:**
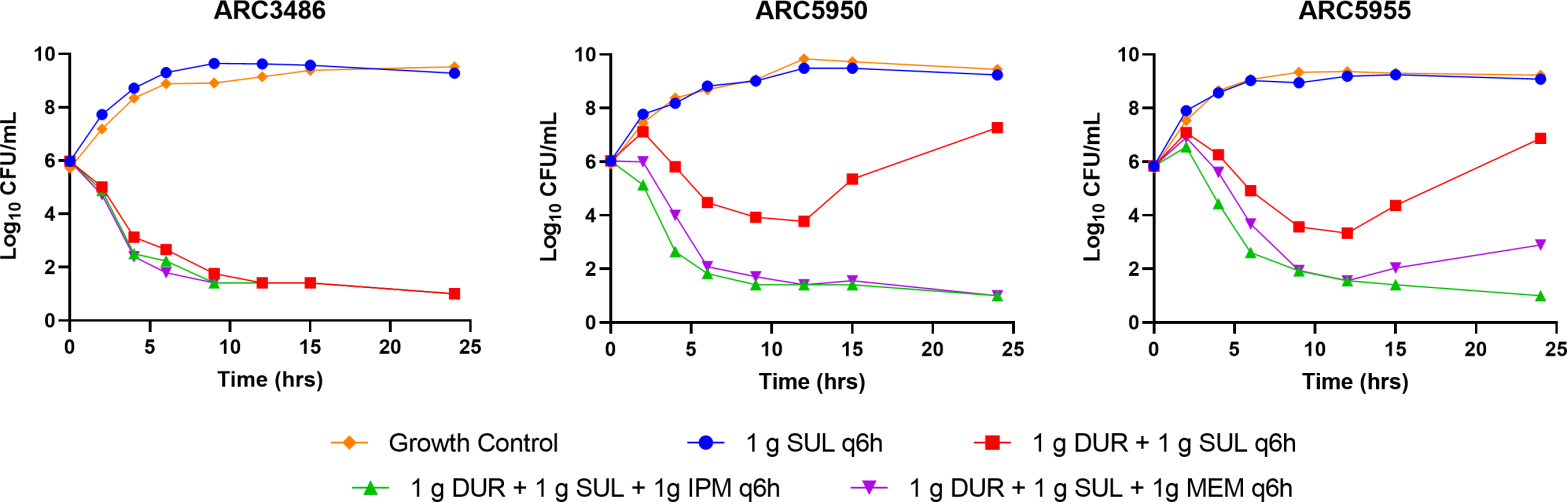
Time course CFU burden vs time for sulbactam 1 g q6h alone and in combination with 1-g q6h durlobactam compared to vehicle control and co-administration of either 1-g q6h of imipenem or 1-g q6h of meropenem against ARC3486, ARC5950, and ARC5955.

Clinical regimens of sulbactam and durlobactam mimicking epithelial lining fluid (ELF) exposures achieved with a 1-g/1-g dose of sulbactam-durlobactam infused over 3 h (20) were evaluated with and without imipenem or meropenem in the HFIM using PVDF cartridges with membrane pore size of 0.1 µm to circumvent the accumulation of β-lactamase enzymes. For the simulation of the 1-g q6h imipenem and meropenem doses, ELF penetration data were utilized to convert plasma concentrations to ELF concentration vs time profiles (21, 22). Simulated sulbactam, durlobactam, imipenem, and meropenem clinical exposures were satisfactorily achieved in all HFIM experiments. Time course CFU burden vs time plots of sulbactam alone, sulbactam + durlobactam, sulbactam + durlobactam + imipenem, and sulbactam + durlobactam + meropenem vs *A. baumannii* ARC3486, ARC5955, and ARC5950 are presented in Fig. 9. Consistent with high observed sulbactam MICs of 16 to 64 µg/mL, treatment with sulbactam alone resulted in net growth of 3.25 to 3.3 log_10_ CFU/mL over 24 h for all three strains. Against *A. baumannii* ARC3486 (sulbactam-durlobactam MIC = 0.5 µg/mL), a net 4.95 log_10_ CFU/mL reduction was observed with and without the addition of each carbapenem. Against the *A. baumannii* PBP3 mutants ARC5955 and ARC5950 with sulbactam-durlobactam MICs of 8 µg/mL, initial bactericidal killing was followed by net regrowth of 1.05 to 1.25 log_10_ CFU/mL by 24 h. Addition of imipenem and meropenem resulted in a net reduction of 5.00 and 2.95 log_10_ CFU/mL, respectively. In all studies, the addition of either imipenem or meropenem resulted in no observed antagonism, and rebounding colonies plated on 3× MIC sulbactam-durlobactam drug plates did not show growth (remained susceptible). Assayed concentrations of sulbactam demonstrated that %T > MIC vs ARC3486, ARC5955, and ARC5950 were 100%, 32%, and 32%, respectively. For durlobactam, AUC_0–24_/MIC ratios of 78.0, 19.5, and 19.5 were estimated vs ARC3486, ARC5955, and ARC5950, respectively.

## DISCUSSION

The objectives of this study were to confirm the PK/PD driver for sulbactam as T > MIC and investigate the PK/PD driver of durlobactam using *in vitro* PK/PD infection model systems. In addition, the exposure magnitudes associated with achieving PK/PD endpoints of 1- and 2-log_10_ CFU/mL reduction over 24 h of treatment were determined in each of the model systems. Two types of cartridges were used in the HFIM systems for the dose fractionation of durlobactam. These cartridges incorporate different type of membrane material, specifically polysulfone and PVDF. The polysulfone cartridges have a 20-kDa molecular weight cut-off, whereas the PVDF cartridges have a larger pore size of 0.1 µm allowing for free diffusion of larger molecules and proteins such as β-lactamases. The PK/PD indices most closely associated with the activity of sulbactam and durlobactam were determined with a sulbactam-susceptible isolate (*A. baumannii* ARC2058) and a sulbactam-resistant, MDR isolate (*A. baumannii* ARC5081), respectively. Dose fractionation studies conducted in the *in vitro* HFIM suggested that the activity of sulbactam was highly correlated to %T > MIC, and AUC_0–24 h_/MIC was highly correlated to the activity of durlobactam vs *A. baumannii* ARC5081 when sulbactam %T > MIC exceeded that required for a 1-log_10_ CFU/mL reduction against susceptible strains. Concentrations of sulbactam observed in the polysulfone cartridges with ARC2058 were less than that predicted suggesting that β-lactamases may have concentrated in the hollow fiber cartridges. In addition, when sulbactam was administered alone vs *A. baumannii* ARC5081, sulbactam concentrations were nearly undetectable by the second dose in the extra-capillary (bacterial) compartment. This model artifact results in an overestimation of the drug exposure needed for efficacy which may not be representative of the infection and distribution dynamics in an *in vivo* setting. Based on this observation, an alternate HFIM cartridge using a larger poor size of 0.1 µm and a one-compartment model were used to determine the magnitude of the PK/PD index of durlobactam associated with its activity in combination with sulbactam vs the MDR *A. baumannii* isolate ARC5081. Using PVDF cartridges with the higher pore size of 0.1 µm, AUC_0–24_/MIC remained as the most correlated index to activity of durlobactam in the HFIM but with lower exposure magnitudes of 13.8 and 24.2 associated with achieving 1-log_10_ and 2-log_10_ CFU/mL reductions, respectively. Interestingly, in the one-compartment model, the activity of durlobactam was highly correlated to a %T > C_T_ of 0.75 µg/mL, utilizing the one strain (ARC5081). Therefore, it remains to be investigated whether the magnitude of the C_T_ threshold or duration for which concentrations need to be maintained are proportional to the MIC as has been shown by other investigators (23, 24). Regardless, in a divided dose setting which is required clinically for sulbactam to meet its %T > MIC target, the *in vitro* pharmacodynamic results suggest that AUC_0–24_/MIC is an appropriate PK/PD index for durlobactam as established in the HFIM. Consistent with the hypothesis that accumulation of β-lactamases in the HFIM could result in higher exposure magnitudes of durlobactam to achieve PK/PD endpoints, lower AUC_0–24_/MIC ratios of 7.6 and 33.4 were determined in the one-compartment model for 1- and 2-log_10_ CFU/mL reductions, respectively. Lower magnitudes were also observed in the HFIM using the larger pore PVDF cartridges with estimates for durlobactam AUC_0–24_/MIC for 1-log_10_ and 2-log_10_ CFU/mL reductions in line with the one-compartment model. Similarly, sulbactam exposure magnitudes *in vivo* were also lower to achieve bactericidal activity with T > MIC of 50% associated with achieving 1-log_10_ CFU reduction in the murine neutropenic thigh and lung model vs ARC3486 (25).

Utilizing the PVDF cartridges, simulated ELF drug concentration vs time profiles associated with 1-g doses of sulbactam, durlobactam, imipenem, and meropenem were used to assess the activity of the combinations against relevant contemporary CRAB isolates in the HFIM. Sulbactam-durlobactam 1 g/1 g was infused over 3 h, and imipenem and meropenem were infused over 1 h every 6 h. These dose and infusion schedules were projected to be associated with achieving clinical efficacy against CRAB isolates with sulbactam-durlobactam MICs ≤4 µg/mL based on the 3-h infusion of 1-g sulbactam q6h exceeding T > MIC (4 µg/mL) for greater than 50% and a durlobactam AUC_0–24_ of 160.4 associated with a 1-g q6h dose (AUC_0–24_/MIC = 40.1). Activity vs ARC3486 (potentiated MIC = 0.5 µg/mL) was robust with bactericidal reduction of bacterial burden observed with sulbactam-durlobactam with and without a carbapenem. At an MIC of 8 µg/mL for both PBP3 mutants (ARC5950 and ARC5955), bacterial counts rebounded to above stasis presumably due to insufficient sulbactam exposure above the MIC (T > MIC less than 50%). Interestingly, the addition of either imipenem or meropenem to sulbactam-durlobactam in this case reduced bacterial counts below 1-log_10_ CFU/mL, suggesting that the addition of a carbapenem against strains just outside of the susceptibility of sulbactam-durlobactam may provide additive benefit. In recent studies detailing the resistance mechanisms of sulbactam-durlobactam resistance in contemporary strains, there was an apparent difference in sulbactam-durlobactam susceptibility relative to the drivers of resistance. For instance, nearly all metallo-β-lactamase-expressing CRAB isolates demonstrated sulbactam-durlobactam MIC values ≥32 µg/mL, whereas sulbactam-durlobactam MIC values for the majority of PBP3 variants were 8 or 16 µg/mL (26), suggesting that mutations to PBP3 do not completely inhibit susceptibility to sulbactam. The extent that carbapenem addition to the clinical regimen of sulbactam-durlobactam provides additive benefit for the treatment of infections caused by PBP3 mutant CRAB isolates merits further investigation.

The time-dependent nature of durlobactam activity and similar PK was favorable for administration of the two agents as a fixed-dose combination with the same dosing regimen. This ultimately provided the opportunity to utilize the AUC/MIC ratio as an exposure target as there would never be an instance where durlobactam would be dosed q24h while sulbactam is administered q6h due to its short half-life. In addition to the HFIM data generated in the present study, *in vivo* studies using multiple CRAB strains exhibiting a broad range of susceptibilities, *f*AUC/MIC demonstrated good correlation coefficients of 0.86 and 0.91 to the activity observed in thigh and lung models, respectively (27). More recently, an additional index was utilized to characterize time-dependent activity of compounds such as sulbactam and durlobactam that demonstrate short half-lives or in instances where maximal activity fails to be clearly defined in the dose-response curve (28, 29). In these studies, AUC_0–24_/MIC was corrected by the dosing interval by multiplying it by 1/tau and plotting the result vs the 24-h change in CFU burden. Fitting the data to a Hill-type model, higher correlations could be obtained with AUC describing the time-dependent PK/PD behavior. A similar investigation was performed with durlobactam utilizing the dose fractionation data from the chemostat model of the present study. This included all the data from the q6h, q12h, and q24h regimens (30). Fitting of the data demonstrated AUC_0–24_ * 1/tau of 6.58 and 23.07 for 1- and 2- log_10_ CFU reductions. In consideration of a q6h dosing interval, these values equate to AUC_0–24_ of 39.5 and 138.4 or AUC_0–24_/MIC of 9.9 and 34.6, respectively (ARC5081 MIC = 4 µg/mL). These values are consistent with mean AUC_0–24_/MIC estimates of 7.6 and 33.4 estimated in the one-compartment model and 13.8 and 24.2 in the HFIM utilizing the larger pore size PVDF membranes.

In conclusion, *in vitro* pharmacodynamic systems demonstrated %T > MIC and AUC_0–24_/MIC to be highly correlated to the activity of sulbactam and durlobactam, respectively, vs susceptible and MDR *A. baumannii* strains including CRAB isolates. PK/PD exposure magnitudes established from the *in vitro* one-compartment model and dose fractionation of durlobactam performed in the HFIM utilizing 0.1-µm pore size cartridges were generally consistent and contrasted with exposure magnitudes derived from HFIM experiments incorporating polysulfone cartridges. Lower than predicted concentrations of sulbactam in the absence of durlobactam may suggest that accumulation of β-lactamase occurs in the extra-capillary space of the cartridges, potentially confounding exposure magnitude estimates. Further HFIM studies using simulated clinical ELF exposures in the PVDF cartridges (0.1-µm pore size) suggest that efficacy may be achieved with a 1-g/1-g q6h regimen of sulbactam-durlobactam against CRAB isolates with MICs ≤4 µg/mL.

## MATERIALS AND METHODS

### Bacterial isolates

All strains with an “ARC” designation are part of the Entasis Therapeutics collection of clinical isolates. All isolates have been previously characterized by whole genome sequencing.

### Antimicrobial susceptibility testing

Broth MIC testing was performed according to the CLSI methodology (31). The recommended quality control bacterial strains *E. coli* ATCC 25922 and *A. baumannii* NCTC 13304 were incorporated into testing according to the CLSI guidelines to assure that there was no variation between test dates [CLSI M100-S27, 2017 (32)]. *A. baumannii* isolates were selected based on their β-lactamase gene content and PBP3 mutations, previously determined by whole genome sequencing, and their range of susceptibilities to sulbactam. Sulbactam-durlobactam MIC testing was performed as a titration of sulbactam in the presence of a fixed concentration of 4 µg/mL durlobactam. MIC testing of sulbactam-durlobactam in the presence of a carbapenem was performed as a titration of a 1:1 ratio of sulbactam:carbapenem in the presence of a fixed concentration of 4 µg/mL durlobactam.

### Hollow fiber infection model vs *A. baumannii* ARC2058 and ARC5081 using polysulfone cartridges

A series of experiments was conducted utilizing an *in vitro* HFIM (33) to investigate the PK/PD index associated with the efficacy of sulbactam alone vs sulbactam-sensitive strain *A. baumannii* ARC2058 and durlobactam when dose fractionated on top of a q6h regimen of sulbactam vs an MDR *A. baumannii* isolate, ARC5081. Steady-state fluctuating free-drug plasma concentrations were simulated in the *in vitro* HFIM to evaluate bacterial response to various sulbactam and durlobactam exposures over a period of 24 h at 35°C. Bacterial colonies grown overnight on blood agar were inoculated in Mueller-Hinton Broth II (MHBII, Sigma-Aldrich, St. Louis, MO) and incubated at 35°C until reaching log phase growth (~1 h). Culture was diluted to reach target inoculum (1 × 10^6^ CFU/mL). In studies incorporating polysulfone cartridges, approximately 15 mL of culture was introduced to the extra-capillary space of the hollow fiber cartridges (Polysulfone catalog no. C2011 FiberCell Systems*,* Inc., Frederick, MD) and were exposed to various dosing regimens of sulbactam via syringe pump (Harvard Apparatus, Holliston, MA). Total system volume including the cartridge and reservoir was approximately 225 mL. Infusions of 1 h were utilized for dose fractionation of sulbactam vs ARC2058 and an initial study with ARC5081 (Fig. 3). Infusions of 3 h were then performed in subsequent experiments to achieve higher exposure magnitudes for sulbactam T > MIC. Sulbactam and durlobactam were eliminated via isovolumetric dilution of the central compartment with the peristaltic pump rate adjusted to eliminate the compounds with a half-life of 2 h. Serial samples were collected to determine drug concentrations and bacterial burden (log_10_ CFU/mL). The 24-h samples were all plated on drug-supplemented plates (3× the MIC) to screen for potential resistant bacterial populations. Against *A. baumannii* ARC2058 sulbactam, regimens of q6h, q12h, and q24h were evaluated spanning a range of AUC_0–24_ from 0 to 441 µg·h/mL, a C_max_ range of 0 to 172 µg/mL, and %T > MIC of 0% to 100%. Against *A. baumannii* ARC5081, a sulbactam q6h regimen was used in all dose arms and was administered to the system via a 3-h infusion with an observed steady-state C_max_ range of 9.5 to 11.1 µg/mL and an AUC_0–24_ range of 209 to 232 µg·h/mL. Durlobactam dose fractionated regimens of q6h, q12h, and q24h were evaluated on top of the q6h regimen of sulbactam spanning a range of AUC_0–24_ from 18.8 to 452 µg·h/mL and a C_max_ range of 1.09 to 119 µg/mL. Serial PK and bacterial (PD) samples were obtained at 0, 1, 2, 3, 4, 6, 9, 12, 15, and 24 h. The 24-h bacterial samples were plated on Mueller-Hinton Agar (MHA, Sigma-Aldrich, St. Louis, MO) supplemented with 4 µg/mL durlobactam and 3× MIC concentration of sulbactam. Plates were incubated at 35°C for 48 h. Colonies that grew on drug plates were evaluated for stable resistance as suggested by a shift in MIC. All PK samples were immediately frozen at −80°C, until assayed.

### Hollow fiber infection model vs *A. baumannii* ARC5081 using PVDF cartridges

Additional HFIM studies were carried in PVDF cartridges vs *A. baumannii* ARC5081 as described above with a targeted inoculum of 1 × 10^6^ CFU/mL. Approximately 5 mL of culture was introduced to the extra-capillary space of the hollow fiber cartridges (PVDF catalog no. C2025 FiberCell Systems*,* Inc., Frederick, MD) and was exposed to various dosing regimens of sulbactam and durlobactam with a 3-h infusion via syringe pump (Harvard Apparatus, Holliston, MA). Total system volume including the cartridge and reservoir was approximately 75 mL. Compounds were eliminated via isovolumetric dilution of the central compartment with the peristaltic pump rate adjusted to eliminate sulbactam with a half-life of 2 h. Serial samples were collected to determine drug concentrations and bacterial burden (log_10_ CFU). The 24-h samples also were plated on drug-supplemented plates (3× the MIC) to screen for potential resistant bacterial populations. A sulbactam q6h regimen used in all dose arms was infused via a 3-h infusion and had an observed C_max_ range of 10.9 to 13.7 and an AUC_0–24_ range of 221 to 236 µg·h/mL. Durlobactam dose fractionated regimens of q6h, q12h, and q24h were evaluated on top of the q6h regimen of sulbactam spanning a range of AUC_0–24_ from 16.0 to 297 µg·h/mL and a C_max_ range of 1.04 to 67.8 µg/mL. Serial PK and bacterial (PD) samples were obtained at 0, 1, 2, 3, 4, 6, 9, 12, 15, and 24 h. The 24-h bacterial samples were plated on MHA (Sigma-Aldrich, St. Louis, MO) supplemented with 4 µg/mL durlobactam and 3× MIC of sulbactam. Plates were incubated at 35°C for 48 h. Colonies that grew on drug plates were evaluated for stable resistance as suggested by a shift in MIC. All PK samples were immediately frozen at −80°C until assayed.

### One-compartment model vs *A. baumannii* ARC5081

A one-compartment *in vitro* infection model was utilized in these studies as described previously (34). This model consisted of a central infection compartment containing growth medium, the challenge isolate, and a magnetic stir bar to ensure the homogeneity of the drug(s) within the compartment. The central infection compartment was set upon a stir plate, at a mixing speed of 125 revolutions per minute, and placed within a humidified incubator set at 35°C. Drug-free growth medium was continuously pumped into the central infection compartment via a computer-controlled peristaltic pump in order to simulate human free-drug plasma concentration-time profiles for both sulbactam and durlobactam. Excess growth medium was simultaneously removed through an exit port and captured in a waste container. *A. baumannii* ARC5081 was aseptically inoculated directly into the central infection compartment. Durlobactam and sulbactam were infused via computer-controlled syringe pumps which allow for the simulation of multiple dosing frequencies, half-lives, and concentrations. Specimens were collected for the enumeration of bacterial density and drug concentration assay directly from the central infection compartment using a sterile syringe and needle through a rubber septum at pre-determined time points.

In these experiments, an initial inoculum of 1.0 × 10^6^ CFU/mL of *A. baumannii* ARC5081 was prepared from a culture grown overnight on trypticase soy agar enriched with 5% sheep blood (BD Laboratories, Franklin Lakes, NJ). Single colonies were taken from the overnight cultures and grown to mid-logarithmic phase in a flask of Mueller-Hinton broth set in a shaking water bath at 35°C and 125 revolutions per minute. The bacterial concentration within the flask of Mueller-Hinton broth was determined by optical density and a previously confirmed growth curve for each challenge isolate. One-milliliter specimens were collected for CFU determination at 0, 2, 4, 8, 12, and 24 h. Each sample was centrifuged, washed, and resuspended with sterile normal saline twice to prevent drug carryover and then cultured onto trypticase soy agar enriched with 5% sheep blood. A small portion of the bacterial sample was plated on to Mueller-Hinton agar plates supplemented with 3× the durlobactam potentiated sulbactam MIC in order to enumerate the sulbactam-durlobactam-resistant subpopulation. Plated samples were incubated at 35°C for 24 h. An intensive PK sampling strategy was utilized over the study duration to ensure the capture of peak (C_max_) and trough (C_min_) concentrations, with time points taken at 1.5, 1, 3, 4, 5, 7.5, 9, 11, and 23 h post treatment initiation. In order to determine the durlobactam exposure range to be utilized in the dose fractionation studies, a series of duplicate dose-ranging studies were completed. *A. baumannii* ARC5081 was exposed to changing concentrations of sulbactam representing free-drug plasma exposures observed following an intravenous dose of 2 g administered q6h and infused over a 3-h duration (35). The 2 g q6h regimen of sulbactam was examined alone and in combination with a range of durlobactam exposures (AUC0-24 ranging from 18.5 to 591 μg.h/mL). An arm with durlobactam alone targeted at a 4 g q6h dose and a no-treatment regimen served as a negative controls. Based upon the results of the dose-ranging studies, a series of duplicate dose fractionation studies were completed using the same *A. baumannii* isolate (ARC5081) at an initial inoculum of 1.0 × 10^6^ CFU/mL. Sulbactam 2-g q6h doses were administered in combination with four durlobactam total daily doses of 0.5, 2, 4, or 8 g (free-drug AUC_0–24_ of 13.9, 55.8, 111, and 222, respectively) fractionated by AUC_0–24_ into regimens administered q6h, q12h, and q24h. Control arms with no treatment and durlobactam administered 4 g q6h served as negative controls. Samples were collected for the enumeration of the bacterial populations at 0, 2, 4, 8, 12, and 24 h and plated on drug-free agar. An intensive PK sampling strategy was utilized over the study duration to ensure the capture of peak (C_max_) and trough (C_min_) concentrations, with time points taken at 1.5, 1, 3, 4, 5, 7.5, 9, 11, and 23 h post treatment initiation. All PK samples were immediately frozen at −80°C until assayed.

### Pharmacokinetic-pharmacodynamic analysis

Pharmacokinetic models were fit to the time vs drug concentration profiles generated in the *in vitro* HFIM and one-compartment model systems using Phoenix WinNonLin 8.2. PK parameter estimates were derived from a one-compartment PK model. The sulbactam or durlobactam AUC_0-24_, C_max_, and the percentage of the dosing interval that drug concentrations were above the MIC or concentration threshold were calculated for each exposure simulated in the dose fractionation studies. A Hill-type model was fit to the pharmacodynamic data generated from the dose fractionation studies using non-linear least squares regression. For the one-compartment model, the relationship between change in log_10_ CFU from baseline at 24 h and durlobactam AUC_0–24_, C_max_, and %T > various concentration threshold values ranging from 0.5 to 2 µg/mL were evaluated to determine the PK/PD index most closely associated with the activity. For the HFIM dose fractionation of sulbactam and durlobactam, the relationships between change in log_10_ CFU from baseline at 24 h and sulbactam or durlobactam AUC_0–24_/MIC, C_max_/MIC, and %T > MIC were evaluated using Hill-type models. Magnitudes associated with stasis, 1-log_10_ and 2-log_10_ CFU reduction from baseline at 24 h as well as the exposure required for an 80% reduction in bacterial burden (EC_80_) were determined from the fitted function. The PD/efficacy results were determined per regimen as the 24-h difference of total microbial population (CFU) between time 0 h and 24 h post initiation of therapy.

### Human ELF exposure time course simulations in HFIM with and without meropenem or imipenem

The inoculum of ARC3486, ARC5955, and ARC5950 was prepared for infection from an overnight blood agar plate culture (Remel, Lenexa, KS). On the day of the experiment, a few colonies of bacteria were inoculated in MHBII (Sigma-Aldrich, St. Louis, MO) and incubated shaking at 35°C until reaching log-phase growth for about 1 h. Cell population density is confirmed by turbidometric assay (OD_600_), and final dilution is adjusted to deliver approximately 5 mL of bacteria into the extra-capillary space of the hollow fiber cartridge to achieve a final bacterial target burden of (1 × 10^6^ CFU/mL). After 1 h, each hollow fiber cartridge (PVDF catalog no. C2025 FiberCell Systems, Inc., Frederick, MD) was exposed to targeted drug concentration vs time profiles of sulbactam or sulbactam-durlobactam (via 3-h infusion) alone and in combination with either imipenem or meropenem (via 1-h infusion). The doses were administered via syringe pump (Harvard Apparatus, Holliston, MA). The system was programmed to produce targeted half-life and infusion duration that would reproduce approximate clinical ELF PK profiles for all tested compounds. One cartridge served as a growth control and received only vehicle (no drug treatment).

The experimental set-up was maintained at 35°C for the duration of the experiment, and the runs were completed in duplicate. Bacterial burden (CFU/mL) was serially assessed by sampling (1 mL) from the hollow fiber cartridge at 0, 3, 6, 9, 12, 15, and 24 h following initiation of therapy. Serial PK samples (200 µL) were also collected at the same timepoints. All PK samples were immediately frozen at −80°C until assayed. PK samples were assayed by LC/MS/MS as detailed in Table S2. Bacterial samples were diluted (serial 10-fold dilutions) and plated on blood agar plates (Remel, Lenexa, KS) and incubated at 35°C overnight before colony-forming units were enumerated visually. The 24-h bacterial samples were plated on MHA (Sigma-Aldrich, St. Louis, MO) supplemented with 4 µg/mL durlobactam and 3× MIC concentration of sulbactam. Plates were incubated at 35°C for 48 h. Colonies that grew on drug plates were evaluated for stable resistance as suggested by a shift in MIC.

### Sulbactam and durlobactam concentration determination

Serial samples collected from the HFIM and one-compartment model over the 24-h time period was assayed by LC/MS/MS (Sciex API 5000) to confirm the targeted concentration-time profile using a qualified non-GLP bioanalytical method (Table S2). Prepared calibration standards in the assay (1, 2.5, 5, 10, 25, 100, 500, 1,000, 5,000, and 10,000 ng/mL) were made by serial dilution in 1:1 MHBII broth:blank plasma prior to processing. Additional samples and standards were prepared in plain MHBII broth for the assay of imipenem or meropenem. Sample aliquots (50 µL) were added in the 96-well plates. Then, 150 µL of 100% acetonitrile and 0.1% formic acid containing 250 ng/mL of carbutamide as an internal standard were added to the plate. Due to instability of imipenem in 100% acetonitrile and 0.1% formic acid, a separate aliquot was added in a 96-well plate containing water and 250 ng/mL carbutamide. Plates were covered and centrifuged at 4,000 × *g* for 5 minutes. Supernatant (100 µL) was transferred to a clean collection plate. Samples were mixed well and analyzed by LC/MS/MS (Sciex API 5000, Analyst v1.6.1); LC/MS/MS instrument parameters are summarized in Table S2. The assay had a lower limit of quantitation (LLOQ) of 1 ng/mL and upper limit of quantitation (ULOQ) of 10,000 ng/mL. Assay performance is summarized in Table S3.

## SUPPLEMENTAL MATERIAL

Supplemental information aac.00312-23-s0001.docx

ASM does not own the copyrights to Supplemental Material that may be linked to, or accessed through, an article. The authors have granted ASM a non-exclusive, world-wide license to publish the Supplemental Material files. Please contact the corresponding author directly for reuse.
