# Supplementary material for: In vitro pharmacokinetics/pharmacodynamics of the β-lactamase inhibitor, durlobactam, in combination with sulbactam against Acinetobacter baumannii-calcoaceticus complex
Source: Antimicrob Agents Chemother. 2023 Dec 11;68(1):e00312-23. doi: 10.1128/aac.00312-23 (PMC10869334; doi:10.1128/aac.00312-23)
Supplement: Supplemental information [file aac.00312-23-s0001.docx]

**Supplemental Information**

**Supplement Information Table 1. PK/PD Summary of Durlobactam Dose Fractionation vs. ARC5081 in HFIM**

| Cartridge: | Polysulfone | | PVSF (0.1 µm pore size) | | One-compartment model |
| --- | --- | --- | --- | --- | --- |
|  | R^2^ | WSSR | R^2^ | WSSR | R^2^ |
| AUC_0-24_/MIC | 0.85 | 59 | 0.96 | 5.2 | 0.613 ^a^ |
| Cmax/MIC | 0.72 | 33 | 0.89 | 15 | 0.507 ^b^ |
| T>[C_T_] (µg/mL) |  |  |  |  |  |
| C_T_ = 0.5 | 0.42 | 100 | 0.62 | 45 | 0.756 |
| C_T_ =0.75 | 0.56 | 83 | 0.71 | 37 | 0.827 |
| C_T_ = 1 | 0.68 | 66 | 0.84 | 21 | 0.788 |
| C_T_ = 2 | 0.77 | 50 | 0.89 | 15 | 0.695 |
| C_T_ = 4 | 0.72 | 58 | 0.80 | 27 | ND |

ND = not determined; ^a^AUC_0-24_; ^b^Cmax

**Supplement Information Table 2. UPLC/MS/MS Instrument Conditions for Sulbactam and Durlobactam Quantitation**

| Instrument | Schimadzu UPLC - Sciex 5000 LC-MS/MS Mass Spectrometer | |
| --- | --- | --- |
| Column | Atlantis T3, 5µ, 50 x 3.0mm | |
| Column Temperature | 35^0^C | |
| Sample Temperature | 10^0^C | |
| Flow rate | 1.200 mL/min | |
| Gradient | Time (min) | %B |
|  | Initial | 2.0 |
|  | 0.3 | 2.0 |
|  | 1.3 | 98 |
|  | 1.75 | 98 |
|  | 1.76 | 2.0 |
|  | 2.00 | Stop |
|  |  | |
| Divert Valve | 0.30 min to Mass Spec  1.80 min to waste | |
| Mobile Phase A | 0.1% formic acid in water | |
| Mobile Phase B | 0.1% formic acid in Acetonitrile | |
| MRM | Source Type Turbo Spray  Polarity: Negative (Positive for Meropenem and Imipenem)  Resolution Q1: Unit  Resolution Q3: Unit  DP -40.00  CXP -15.00  CE -27.00  IS: -4000.00  TEM: 600.00  GS1: 60.00  GS2: 55.00  CAD: 12.00 | |
| Injection volume | 1.0 µL | |

MRM Transitions:

| Compound ID | Mode | Q1 | Q3 | DP | CE | CXP |
| --- | --- | --- | --- | --- | --- | --- |
| durlobactam | Negative | 276.10 | 96.1 | -40 | -27 | -15 |
| sulbactam | Negative | 232.1 | 139.9 | -58 | -18 | -19 |
| imipenem | Positive | 300.1 | 141.9 | 56 | 39 | 20 |
| meropenem | Positive | 384.1 | 141.0 | 25 | 21 | 10 |
| Carbutamide (IS) | Negative | 270.00 | 171.00 | -55 | -25 | -10 |

**Supplement Information Table 3. LC/MS/MS Assay Performance Summary**

| Compound ID | LLOQ  (ng/mL) | ULOQ  (ng/mL) | Standards  % recovery (SD) | QC  % recovery (SD) |
| --- | --- | --- | --- | --- |
| durlobactam | 1.0 | 10000 | 99.3% (8.5%) | 95.3% (6.7%) |
| sulbactam | 1.0 | 10000 | 98.2% (11.3%) | 96.1% (6.3%) |
| imipenem | 1.0 | 10000 | 100.0% (7.2%) | 94.6% (8.5%) |
| meropenem | 1.0 | 10000 | 97.5% (6.8%) | 99.3% (7.4%) |

LLOQ = lower limit of quantitation

ULOQ = upper limit of quantitation

SD = standard deviation

**Supplement Figure 1. Observed vs. Predicted Sulbactam (A) and Durlobactam (B) Concentrations of in the HFIM Using PVDF (0.1 µm pore size) cartridges.**

**(A)**

**(B)**

**Supplement Figure 2. Observed vs. Predicted Sulbactam (A) and Durlobactam (B) Concentrations in the One-Compartment Model.**

**(A)**

**(B)**

**Supplement Figure 3. Predicted vs. Observed Simulated ELF Concentrations of Sulbactam (A), Durlobactam (B), Imipenem (C), and Meropenem (D) in HFIM (PVDF 0.1 um cartridges)**

**(A)**

**(B)**

**(C)**

**(D)**
